# Perspectives of hyperpolarized noble gas MRI beyond ^3^He

**DOI:** 10.1016/j.jmr.2012.11.014

**Published:** 2013-04

**Authors:** David M.L. Lilburn, Galina E. Pavlovskaya, Thomas Meersmann

**Affiliations:** University of Nottingham, School of Clinical Sciences, Sir Peter Mansfield Magnetic Resonance Centre, Nottingham NG7 2RD, United Kingdom

**Keywords:** ^129^Xe, Xenon-129, Xe-129, ^83^Kr, Krypton-83, Kr-83, Hyperpolarization, Hyperpolarized, Spin polarization, Spin-exchange optical pumping, Chemical shift selective, Nuclear electric quadrupole moment, Quadrupolar relaxation, NMR spectroscopy, Pulmonary MRI, Lung pathology, Biosensor, Molecular imaging, Remote detection, Flow, Diffusion, Gas phase, Transport weighted, Combustion, Surface sensitive, MRI contrast, Porous materials

## Abstract

Nuclear Magnetic Resonance (NMR) studies with hyperpolarized (hp) noble gases are at an exciting interface between physics, chemistry, materials science and biomedical sciences. This paper intends to provide a brief overview and outlook of magnetic resonance imaging (MRI) with hp noble gases other than hp ^3^He. A particular focus are the many intriguing experiments with ^129^Xe, some of which have already matured to useful MRI protocols, while others display high potential for future MRI applications. Quite naturally for MRI applications the major usage so far has been for biomedical research but perspectives for engineering and materials science studies are also provided. In addition, the prospects for surface sensitive contrast with hp ^83^Kr MRI is discussed.

## Graphical abstract

**Graphical abstract:**

**Figure:**
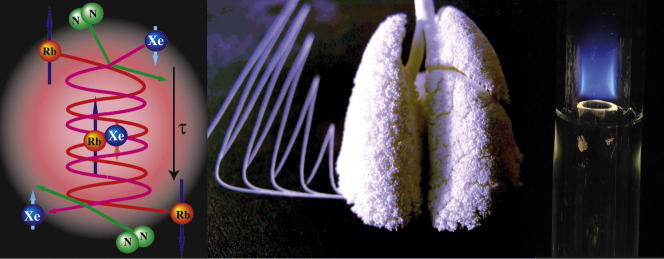

**Highlights:**

► Advances in hyperpolarization have reduced the SNR gap between ^3^He and ^129^Xe. ► ^129^Xe offers additional information useful in materials and biomedical sciences. ► Non-invasive techniques with ^129^Xe probe lung morphometry and offer new biomarkers. ► ^83^Kr with a nuclear electric quadrupole moment provides surface sensitive contrast.

## 1 Introduction

Although magnetic resonance imaging (MRI) of the gas phase is possible without the use of hyperpolarized (hp) spin states [1], the density of gases at ambient pressure and temperature is typically reduced by about three orders of magnitude compared to the respective condensed phase. This significantly lowers Nuclear Magnetic Resonance (NMR) signal intensities and limits magnetic resonance imaging (MRI) resolution as the MRI experiments require gases with high gyromagnetic ratio, *γ*, high spin concentrations, and shorter longitudinal (*T*_1_) relaxation times (to allow for rapid signal averaging). Hp spin states, on the other hand, can enhance the NMR signals by many orders of magnitude compared to thermally polarized states and enable gas phase MRI of both dilute spin systems and nuclei with low gyromagnetic ratios. Since the hyperpolarization is almost always produced outside the MRI detection region, the hp gas typically requires some form of transport from the hyperpolarizer to the detection zone and sufficiently long relaxation times are needed to sustain the generated hyperpolarized state until NMR signal detection has occurred. There is no disadvantage from slow *T*_1_ relaxation in hyperpolarized MRI because signal averaging is not based on relaxation recovery but on renewed delivery of hyperpolarized species for every scan. Unfortunately, most molecules experience fast relaxation in the gas phase due to spin–rotation interactions. A noticeable exception is the group of mono-atomic noble gases where spin–rotation relaxation only occurs during short-lived interaction with other atoms [2]. Therefore *T*_1_ times of many hours and even days can be possible unless additional relaxation mechanisms are present [2–5].

To date, the most widespread and successful MRI applications of hp noble gases utilize the isotope ^3^He (spin *I* = ½, NMR frequency 75.905 MHz at 2.35 T) for preclinical and clinical studies of pulmonary pathophysiology. A review of the successful applications with hp ^3^He MRI would exceed the purpose of this paper and is therefore best left to the specialists in this field (see for instance [6–8] for previous reviews). Furthermore, the main supply source for ^3^He is tritium decay in nuclear (fusion) warheads with no viable current alternative in sight. The very high demand for this isotope for many types of applications has therefore led to a ^3^He supply crisis as evidenced by US congressional hearings [9]. The best remedies to this problem for the MR community may be rigorous ^3^He recycling whenever possible and the exploration of alternative techniques.

Hp ^129^Xe, in particular, has found applications in medical research but, unlike ^3^He, also has been used in materials science, chemistry and related disciplines where the large ^129^Xe chemical shift range is exploited for NMR spectroscopy [10–17]. Therefore, hp ^129^Xe MRI is at a stimulating interface between physical and biomedical sciences and this article focuses on actual and prospective hp ^129^Xe MRI methods in many research fields. In addition, hp ^83^Kr MRI which exploits the nuclear electric quadrupole moment of this noble gas isotope for surface sensitive contrast will also be covered.

## 2 Hp ^129^Xe MRI

Next to ^3^He, the most prominent noble gas isotope for hp gas phase MRI is ^129^Xe that has already found its way into preclinical and clinical usage. Indeed, the first noble gas lung MRI reported by Albert et al. in 1994 utilized hp ^129^Xe [18]. The isotope ^129^Xe has a nuclear spin *I* = –1/2 with an NMR frequency of 27.6 MHz at 2.35 T magnetic field strength (i.e. 100 MHz ^1^H frequency) for elemental xenon at ambient pressure and temperature. Xenon is a renewable resource obtained from air liquefaction with a natural abundance of 26.4% ^129^Xe and isotopic enrichment is available at affordable costs (i.e. currently US$ 200–250 per liter gas at ambient pressure and temperature, depending on the fluctuating actual market and specific offers. Xenon gas with natural abundance isotope distribution typically costs around US$ 10–12 per liter gas). The signal intensity of ^129^Xe falls short compared to that of hp ^3^He because of the 2.74 times larger gyromagnetic ratio of ^3^He and because of the high spin polarizations routinely obtained with ^3^He that exceeded those typically achieved for ^129^Xe.

**Fig. 1:**
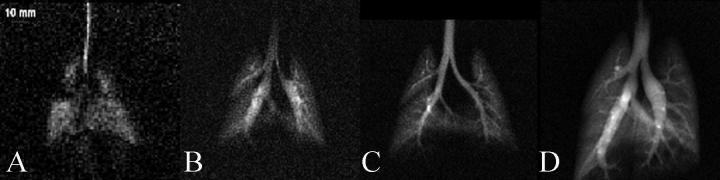
Evolution of hp ^129^Xe image resolution over a decade. (A) Early image of a rat lung from 1998 with in-plane resolution 0.84 × 0.84 mm^2^ and SNR of ∼3. (B–D) Progressively better image quality as polarization, gas delivery and MR acquisition techniques continue to improve. (D) Image from 2007 with resolution 0.31 × 0.31 mm^2^ and an SNR of ∼20. Reprinted with permission from Driehuys et al. Toxicol. Pathol. 2007; 35:55. © SAGE Publications.

For a hyperpolarized spin system, the NMR signal intensity is proportional to the square of the gyromagnetic ratio assuming identical conditions with respect to the polarization value *P*, magnetic field strength *B*_0_, spectral width, and NMR hardware. However, the signal losses due to electrically conducting, whole body sized media at typical MRI field strengths (1.5 T and above) increases with higher frequencies. For whole body hp ^129^Xe and hp ^3^He MRI applications one therefore usually assumes only a linear dependence of the MR signal intensity on the gyromagnetic ratio. In addition, depending on the particular application, the disadvantage for ^129^Xe and its lower resonance frequency may be further reduced at higher field strengths because its smaller gyromagnetic ratio means less shortening of the $T_{2}^{*}$ values (generally caused by magnetic susceptibility effects in heterogeneous media such as the lungs). In addition, due to ever increasing progress in spin exchange optical pumping (SEOP), very high ^129^Xe polarization values have now been reached at high production rates [19–23]. This has ultimately reduced the SNR gap between ^3^He and ^129^Xe, directly improving the temporal and spatial resolution of hp ^129^Xe imaging. Optimization of hardware and MRI protocols leads to further advances in the quality of the MR images. See Fig. 1 for an example of the ‘evolution’ of hp ^129^Xe lung MRI over the past two decades [24].

## 3 Hp noble gas production

A hyperpolarized spin state is simply a state at very low spin temperature that is not in a thermal equilibrium with the (motional) temperature of the sample. Low spin temperature leads to high population of the ground state and thus high magnetization of the spin ensemble that results in very high NMR signal intensity. This state eventually returns to the thermal equilibrium temperature (i.e. depolarizes). Therefore, *T*_1_ relaxation needs to be slow enough to preserve the state for sufficient periods of time. The hyperpolarized state can, in principle, be generated through rapid heating of a sample from the thermal equilibrium at very low temperatures (*T* ≪ 1 K) [25]. Experimentally less demanding, all noble gas isotopes with non-zero nuclear spin can be hyperpolarized through spin exchange optical pumping (SEOP) using alkali metal vapor [26]. Although SEOP is typically performed at temperatures above 350 K and under high power laser irradiation, it selectively reduces the temperature of the nuclear spin to values far below 1 K. For this to be useful for MRI, the reactive alkali metal (typically rubidium) needs to be removed before the hp gas is transferred for MRI detection [27,28]. Slow *T*_1_ relaxation is needed to preserve the low spin temperature that is not in a thermal equilibrium with the molecular environment. The nuclear spin polarization of a hyperpolarized sample is best determined through the signal enhancement factor obtained from comparison of the associated hp NMR signal with that of a thermally polarized sample at otherwise identical – or at least at comparable – conditions. At ambient temperatures and high magnetic field strengths, the thermal spin polarization can be straightforwardly calculated using:

(1) $$P_{\mathrm{therm}}=\frac{|\gamma |\hbar B_{0}}{3k_{B}T}(I+1)$$

where *I* is the nuclear spin, *γ* is the gyromagnetic ratio, *k_B_* is the Boltzmann constant, and $\hbar =\frac{h}{2\pi }$ is the Planck constant [29]. The polarization *P_hp_* of the hp sample is simply the product of *P_therm_* and the SEOP enhancement factor. SEOP can be performed either in a stopped flow mode [27,30,31] or in a continuous flow mode [20]. Typically SEOP uses a mixture of gases that contain xenon (or krypton) in low concentrations and N_2_ and helium (^4^He) in abundance. Though low noble gas concentration reduces the MR signal intensity, hp ^129^Xe can be concentrated through cryogenic separation [19,20,23,32,33].

Many advances have been made in continuous flow SEOP leading to very high spin polarization values at high production rates [19–23,32,34,35]. At the pinnacle of current technology, Hersman and co-workers have developed a fully automated SEOP system with cryogenic separation that is capable of producing multiple liters of hp ^129^Xe per hour with a spin polarization *P_hp_ *= 50% [19,36]. Batch mode SEOP, as a potential low cost alternative, is being further developed using various approaches by other groups [30,31]. For example high noble gas concentration at low pressures in batch mode SEOP has been recently explored to bypass the need for cryogenic separation [31]. This method produced the equivalent of hp ^129^Xe gas with *P_hp_ *= 14% at a rate of 1.8 cm^3^/min using only 23 W of laser power. For hp ^83^Kr, where cryogenic separation is not feasible due to rapid quadrupolar relaxation in the frozen state, the method allowed for *P_hp_ *= 3% at a rate of 2.0 cm^3^/min.

For very specialized applications, it is also possible to hyperpolarize ^129^Xe together with a reactive gas. This has been demonstrated in SEOP of CH_4_–Xe mixtures that served as fuel for hp ^129^Xe MRI of combustion [37]. Methane as a saturated hydrocarbon compound shows little affinity to react with rubidium under SEOP conditions. The polarization obtained in a 5% Xe, 10% N_2_, and 85% CH_4_ mixture was *P_hp_ *= 7% in continuous flow mode at 40 cm^3^/min and *P_hp_ *= 40% in batch mode SEOP.

One crucial element in the improvements of SEOP systems are the many advances made in solid-state laser technology. Line-narrowed laser output at growing power levels becomes increasingly available and affordable [38]. Furthermore, an alternative methodology of potential interest for hp noble gas MRI has recently been explored. Dynamic nuclear polarization (DNP) at 1.2 K was reported as a new approach to generate hp ^129^Xe state at potentially high volumes [39]. Whatever methodology will ultimately be the most successful, the proliferation of techniques to conveniently and inexpensively polarize noble gases appears likely. One should therefore expect for hp noble gas MRI to move beyond its current usage limited to highly specialized research facilities.

## 4 Hp ^129^Xe MRI – Gas phase perspectives

### 4.1 Hp noble gas phase imaging in vivo

**Fig. 2:**
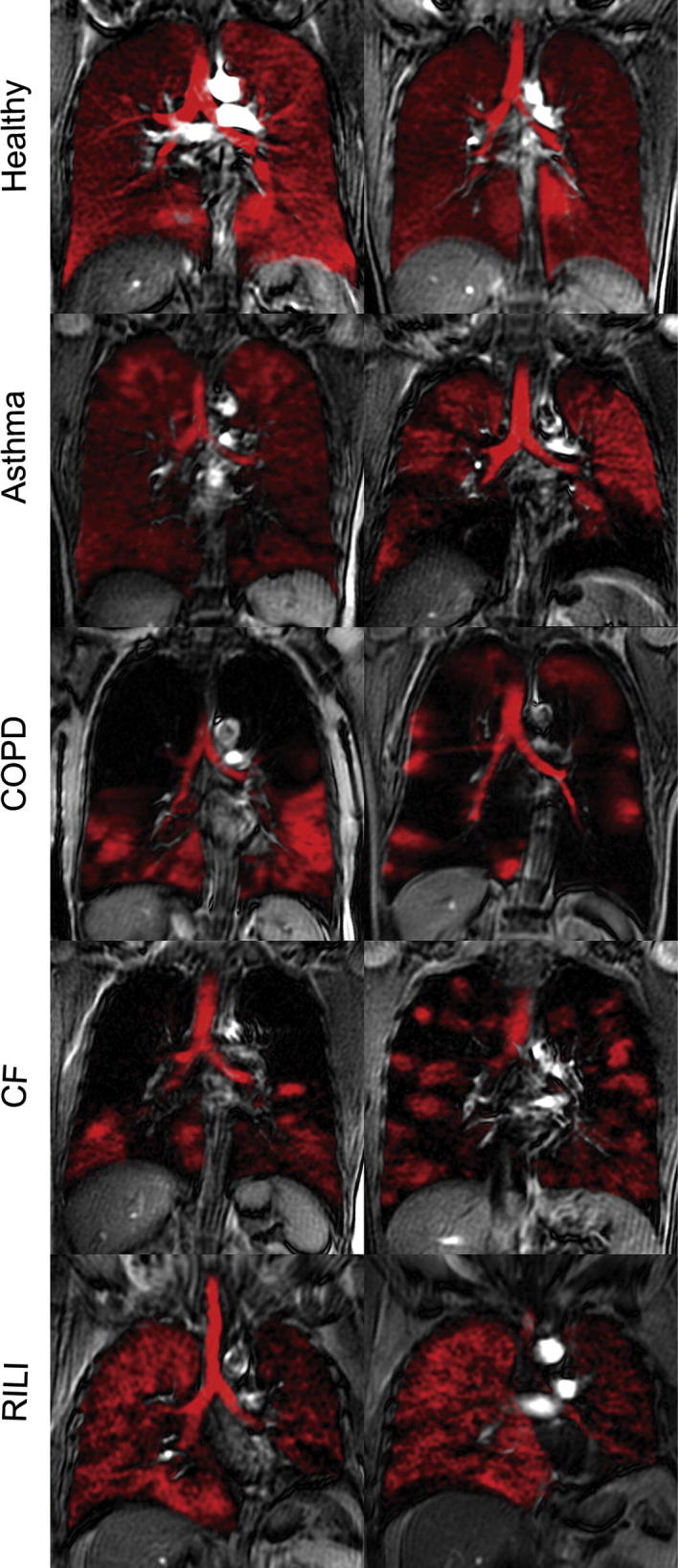
Hp ^129^Xe slice selective coronal MR images (in red) overlayed onto corresponding proton thoracic images from healthy volunteers and subjects with asthma, chronic obstructive pulmonary disease (COPD), cystic fibrosis (CF) and radiation-induced lung injury (RILI). Reprinted with permission from Shukla et al. Acad. Radiol., 2012; 19:944, © 2012 Elsevier. (For interpretation of the references to color in this figure legend, the reader is referred to the web version of this article.)

Possibly the most useful applications of simple spin density gas phase imaging of hp noble gases are in lung functional studies. The clinically most relevant parameter that can be garnered from static pulmonary ventilation scans are ventilation defects [40]. In patients with chronic obstructive pulmonary disease (COPD) or asthma it is possible to monitor the evolution of these defects as the diseases progress over time during clinical, longitudinal studies. It is also possible to observe the response to airway hyperresponsiveness tests in asthma [41]. Effective ventilation deduced by hp MRI *in vivo* has been shown to correlate with spirometry data for patients in health and disease [40,42]. However, although the hp noble gas ventilation images may appear dramatic when displaying larger unventilated areas in lungs it should be noted that this might not be necessarily specific to one disease pathology, rather they reveal the extent and severity of ventilation defects that may be common in many conditions (Fig. 2, [43]).

Safe *in vivo* delivery of hp noble gases merits special mentioning. In general, static ventilation imaging is performed during a simple breath hold after inhalation of the gas mixture containing a known volume of hp gas. Oxygen, can be added to the hp gas for inhalation but paramagnetic O_2_ also leads to an increase in relaxation, for instance the *T*_1_ value drops to approximately 15 s for ^129^Xe in breathable mixtures containing 20% O_2_ [44]. Special care should be taken as xenon becomes a general anesthetic when its alveolar concentration is in the realm of 70% [45]. However a 70% mixture of xenon with 30% N_2_, inhaled for a single breath-hold of 20–40 s, will usually only result in an alveolar concentration of xenon ≈ 35% [46]. Moreover, it has been recently reported that 3–4 repeated inhalation cycles with undiluted one liter boluses of hp ^129^Xe are well tolerated in patients with mild to moderate COPD [47].

**Fig. 3:**
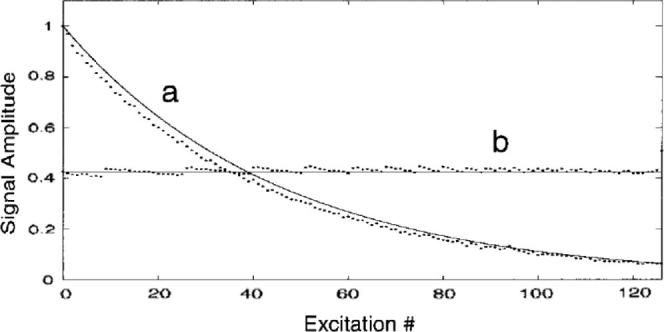
Comparison of signal amplitude between two trains of 127 pulses using constant flip angle (FLASH) of 12° (a) and variable flip angle (VFA) (b) methods. Experimental data are represented by the dots, while the solid lines represent theoretical predictions. Reprinted with permission from Zhao et al. J. Magn. Reson. Ser. B, 1996; 113:180. © 1996 Elsevier.

The most common *in vivo* hp noble gas imaging protocols are still using the concept of FLASH (Fast-Low-Angle-Shot) as their core. Variable flip angle (VFA) MRI sequences, first developed by Zhou et al. [48], are based on an innovative concept that makes full use of the entire hp spin state and therefore lead to improved MR image quality. VFA results in constant signal amplitude (assuming the absence of noticeable *T*_1_ relaxation) until the hp state is completely ‘used up’ (Fig. 3) [48]. Although this methodology has rarely been used for MRI of lungs to date, as it requires careful calibration of the rf pulse power, it can be tremendously beneficial for experiments where low signal intensity is a concern [49],

Technological developments in hardware, computing and image reconstruction might lead to orders of magnitude faster data collection and processing compared to the first *in vivo* attempts. Improvements utilizing echo planar imaging (EPI) and spiral imaging acquisition schemes are already in place for dynamic ventilation imaging with hp ^3^He, however spatial resolution is usually sacrificed for speed. Three-dimensional (3D) dynamic imaging with hp ^3^He within one breath-hold has also been reported [50]. These improvements might be translated to other hp noble gases (^129^Xe, ^83^Kr) given that sufficient advances in SEOP of these species will be achieved.

### 4.2 Dispersion and velocimetry in the gas phase

**Fig. 4:**
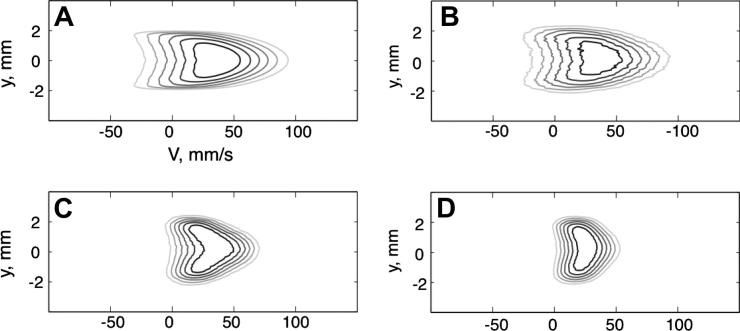
Joint spatial–velocity images of xenon Poiseuille flow in a pipe (id = 4 mm, Δ_Xe_ = 4.5 mm^2^/s, *V*_ave_ = 20 mm/s). The x-axis represents axial velocity (i.e. in z-direction) while the y-axis represents spatial location across the pipe with 0 mm referring to the pipe center. The spectral window in the flow-encoding dimension was kept constant. (A) Computer simulation with Δ = 10 ms. (B) Experiment with Δ = 10 ms. (C) Experiment with Δ = 60 ms. (D) Experiment with Δ = 130 ms. Adapted figure, printed with permission from Kaiser et al. J. Magn. Reson. 2001; 149:145. © 2001 Elsevier.

NMR and MRI velocimetry methods have been extensively reviewed [51]. In principal, the methods can be translated directly to study gas phase flow and dynamics though experiments must be designed with consideration to the specific requirements for gas phase measurements. In non-turbulent flow of liquids, the coherent motion dominates, while contributions from the stochastic dispersion (i.e. diffusion driven) term are negligible. In flowing gases however, stochastic terms may be on the same order of magnitude as the coherent terms arising from the flow. As shown in Fig. 4, this can lead to a strong interplay between coherent flow and Brownian motion depending on the time Δ between the gradient pulses used for displacement encoding.

Whilst at shorter Δ times xenon displaces as predicted numerically (Fig. 4a and b), velocity profiles of xenon for longer Δ times manifest temporal (i.e. Δ dependent) progression of molecular displacements [52]. As Δ becomes longer, dispersion averages the radial dependence of the coherent displacements and results in velocity profiles as displayed in Fig. 4c and d. Therefore, special care needs to be taken in choosing NMR parameters during flow experiments to account for these averaging effects. Nonetheless flow and dispersion can still be probed at a wide range of temporal and spatial scales [51] leading to valuable information in many applications. A novel example is the measurement of gas flow within a flame using a continuous flow of a CH_4_–hp ^129^Xe fuel mixture. MRI of the entire flame region is possible due to the combustion resistance of the ^129^Xe hyperpolarized state [37]. Velocimetric measurements in lungs are also feasible but are experimentally demanding since they cannot be performed in a continuous flow mode. However, some examples using ventilation synchronized measurements have been reported with hp ^3^He [53].

### 4.3 Apparent diffusion coefficient (ADC) measurements *in vivo*

As detailed in the velocimetry section, the results of gas phase pulsed field gradient (PFG) flow measurements may display a dependence upon Δ (i.e. the time between gradient pulses used for displacement encoding). This Δ dependence is due to the interplay of flow and diffusion driven dispersion. Even in the absence of flow, pure diffusion measurements can display a Δ dependence if the gas is contained in a porous medium. For sufficiently short Δ times, the result of the PFG experiments will measure unrestricted diffusion and therefore the same diffusion constant *D_o_* as in the free gas. As Δ becomes longer, the mean displacement of the gas will be hindered by the pore walls, resulting in a reduced apparent diffusion coefficient (ADC).

Diffusion of hp gases in lungs is restricted by alveolar walls and ADC measurements can therefore provide valuable information about lung morphometry [54,55]. Work with ^3^He (binary diffusion coefficient of dilute ^3^He in air ($D_{{}^{3}\mathrm{He}-\mathrm{Air}}=0.86\;{\text{cm}}^{2}/\text{s}$) [56]) has shown that in cases of alveolar destruction such as in emphysematous disease the ADC becomes elevated [57,58]. The ADC measurements for ^129^Xe ($D_{{}^{129}\mathrm{Xe}-\mathrm{Air}}=0.14\;{\text{cm}}^{2}/\text{s}$ [56]) correlate with those for ^3^He [59] with ADC values elevated in human COPD phenotypes [60]. Recently, it has been found that ^129^Xe ADC values may actually correlate better than ^3^He ADC with other lung function testing methods. This may be possibly due to the lower rate of diffusion of xenon leading to less contamination through collateral ventilation from neighboring alveoli [61]. Note, that the ^129^Xe self-diffusion coefficient is six times smaller than that of ^3^He therefore larger field gradients are required to perform the ADC measurements on similar ^3^He time scales. This puts a strain on the hardware safety requirements, however experimental strategies have been proposed to circumvent this problem [62].

## 5 Hp ^129^Xe MRI – Dissolved phase perspectives

### 5.1 The ^129^Xe chemical shift

As ^3^He has a negligible chemical shift and low solubility, its dissolved phase, if any, does not bring any additional information. The situation is different for xenon. Due to its large compressible outer electron shell, ^129^Xe exhibits a significant chemical shift when placed into different chemical environments as compared to the gas phase. The ^129^Xe NMR chemical shift range is just below 300 ppm for the various materials and solvents that may absorb the xenon atoms [11,12,15,16]. Note, that ^129^Xe NMR signal in the bulk gas phase approximated to zero pressure is typically referenced with 0 ppm and the shift increases by about of 0.6 ppm/bar in pure xenon gas at ambient temperature and pressure conditions close to ideal gas behavior. There is an extensive literature covering hyperpolarized ^129^Xe NMR spectroscopy in addition to work with thermally polarized ^129^Xe that utilizes the chemical shift as a ‘spy’ for the environment of the xenon atoms. However, with the recent advances in hyperpolarization of this nucleus, the interrogation of dissolved xenon chemical shift has excellent perspectives for MRI applications in materials science and biomedical studies.

### 5.2 Transport weighted MRI contrast

**Fig. 5:**
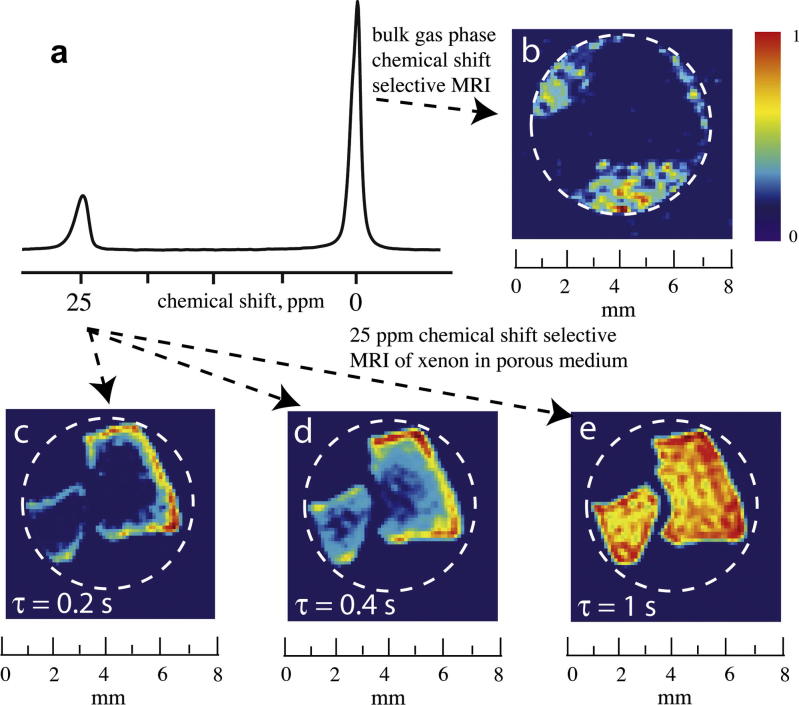
(a) NMR spectrum of hyperpolarized ^129^Xe from a sample that contains gas phase xenon (close to 0 ppm depending on pressure) and xenon occluded within aerogel fragments (around 25 ppm). (b) 2-D slice of 3-D chemical shift selective MRI of the bulk gas phase (0 ppm) surrounding the aerogel fragments. (c–e) 2-D slice of 3-D chemical shift selective MRI using the 25 ppm signal with rising recycle delay times *τ* that lead to an increasing penetration of the hp ^129^Xe into the material. High signal intensity indicates a high concentration of hp ^129^Xe within the aerogel fragments. Inspection of (c–e) reveals that the penetration into the aerogel fragments is strongly anisotropic due to reduced flow between the particles. Adapted figure, printed with permission from Kaiser et al. P. Ntl. Acad. Sci. USA. 2000; 97:2415-6. © 2000 National Academy of Sciences, USA.

^129^Xe chemical shift selective imaging can be used to visualize the effects of gas transport in porous media [63,64]. In conventional MRI, the variation of the recycle delay can lead to *T*_1_ relaxation weighted contrast. In hp MRI, the variation of recycle delay may produce a gas transport weighted contrast if hp ^129^Xe is continuously delivered. The gas is hyperpolarized outside the superconducting magnet and its transport into the sample through flow and diffusion will take time. After a 90° excitation pulse, all hp ^129^Xe within the detection region has been depolarized and the following scan will only detect any signal if the recycle delay is long enough to permit for renewed hp ^129^Xe delivery. This allows for the unique transport weighted contrast that provides a ‘snapshot’ of the gas penetration into porous samples as shown in Fig. 5. Note that the xenon concentration in the sample is constant in time but the ‘concentration’ of the hp nuclear spin state is time dependent. The application of depolarizing radiofrequency (RF) pulses requires that new hp gas is delivered into the material during the recycle delay. At constant recycle delays, a steady state is generated that can be imaged [64].

## 6 ^129^Xe chemical shift in lungs

**Fig. 6:**
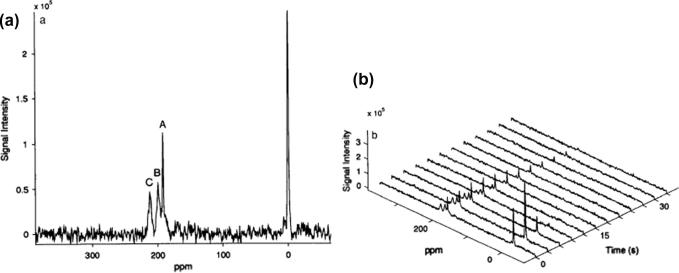
One of the earliest hp ^129^Xe spectra and washout dynamics from the rat pulmonary system. (a) Hp ^129^Xe spectra at *t* = 0 after inhalation of the last xenon bolus. Peak A, B and C at 191, 199, 213 ppm relative to the gas phase peak at 0 ppm attributed to the blood plasma, lung tissue and red blood cells respectively. (b) Temporal dynamics on hp ^129^Xe washout with the combined effects of ventilation, RF depletion and longitudinal relaxation. Reprinted with permission from Sakai et al. J. Magn. Reson. Ser. B, 1996; 111:301. © 1996 Elsevier.

The chemical shift of ^129^Xe is also very useful for pulmonary MRI where continuous flow hp ^129^Xe transport is replaced by usage of the breathing cycle for delivery. When coupled with xenon’s high solubility, it is possible to record a distinct signal arising from xenon atoms associated only with parts of lungs where xenon dissolves, i.e. lung tissue and its components. The first *in vivo* hp ^129^Xe spectra showed four resolved peaks, the initial one located at the gas phase set to 0 ppm and three other peaks were found at 191, 199 and 213 ppm and were attributed to ^129^Xe dissolved in blood plasma, lung tissue and red blood cells (RBCs), respectively (Fig. 6) [65]. The longitudinal relaxation of the peaks associated with the dissolved phase was found to be on the order of seconds thus allowing for the possibility to image xenon incorporated into the tissue components separately from the gas phase [66].

Chemical shift selective MRI of dissolved xenon in lungs is facilitated by the significant frequency shift between ^129^Xe in the gas phase (around 0 ppm) and in the dissolved phase (191–213 ppm) [67]. Unfortunately, xenon in the dissolved phase constitutes only about 1–2% of the total inhaled xenon. Therefore, the associated hp ^129^Xe signal intensity arising from the dissolved phase is fairly weak. Therefore, Fig. 6 does not reflect the true intensity of the gas phase peak because the excitation frequency was selected for the 200 ppm region. If full broadband excitation would be applied, the gas phase peak should be about 50–100 times stronger than the dissolved signal. However, the dissolved phase xenon is constantly replenished from the alveolar gas phase through rapid diffusive exchange. Thus, chemical shift selective excitation of the dissolved phase (i.e. that does not depolarized the hp ^129^Xe in the gas phase) allows for signal averaging with very short delay times in the millisecond regime. Fujiwara and coworkers have demonstrated the use of continuous delivery of hp gas in the mouse lung as a method to enhance the dissolved phases signal [68,69]. Single breath-hold and chemical shift selective three-dimensional MRI of the dissolved phases in human volunteers with reasonable spatial resolution have also been reported [70,71].

**Fig. 7:**
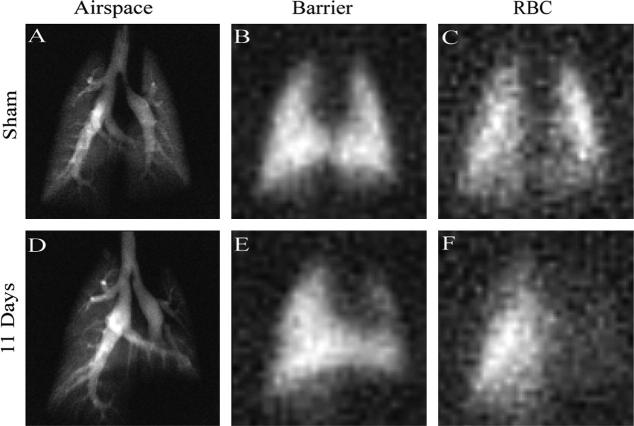
Inhaled hp ^129^Xe images from saline treated rat (sham) and bleomycin treated rat (fibrosis model). Images (A–C) from sham instillation of saline into the left lung. (A) Gas phase ^129^Xe image from the airspaces. (B) Tissue phase ^129^Xe image from the lung parenchyma. (C) Red blood cell (RBC) phase ^129^Xe image. (D–F) Comparable images 11 days after bleomycin instillation into the left lung with D, E and F representing the gas phase, tissue phase and RBC phase images respectively. Note that the tissue images closely match the gas phase images in both rats whilst the RBC phase images show almost absent uptake of ^129^Xe by the RBCs in the bleomycin treated lung in the time-frame of the MRI experiment as compared to the sham treated rat, indicating thickening of the alveolar membrane (fibrosis). Reprinted with permission from Driehuys et al. P. Ntl. Acad. Sci. USA, 2006; 103:18280. © 2006 National Academy of Sciences, USA.

This concept can be used for new physiological measurements that probe gas transfer in lungs using xenon as a surrogate for oxygen and may be helpful for early diagnosis of interstitial lung diseases such as idiopathic pulmonary fibrosis (IPF). Due to a thickening of the lung parenchyma that separates the alveolar space from the blood, gas exchange is reduced in these diseases and gas transport requires longer time periods. Driehuys et al. explored the exchange between the alveolar membrane and capillary blood using a technique called *xenon alveolar capillary transfer imaging* (XACT) [72]. The technique uses chemical shift selective separation between tissue and blood dissolved hp ^129^Xe utilizing the 14 ppm difference between the two dissolved states. The slowed gas transfer from the alveoli to the blood can be visualized with hp ^129^Xe if short recycle delays are used as shown in Fig. 7.

The underlying concept of XACT is chemical shift selected recovery of the hp ^129^Xe signal. This method has been explored by Butler and co-workers to measure surface area to volume ratios (*S*_A_/*V*_gas_) in a variety of porous media and has been applied later in a non-spatially resolved manner to study morphometry of healthy human lungs *in vivo* [73,74]. After a series of selective 90° pulses has destroyed the dissolved phase magnetization and created an initial zero point, the increase of the dissolved phase signal is recorded as a function of time. Assuming that one dimensional diffusion drives signal growth of the dissolved phase one can deduce the *S*_A_/*V*_gas_ in lungs from the dissolved phase to gas phase signal ratio. Recently, this model was refined with lung blood flow corrections and was used to determine additional parameters including alveolar septal thickness (*h*) [75]. The surface area to volume ratio was found to decrease in healthy subjects with increasing inhalation volumes as expected and was noted to be lower in patients with COPD, indicating airspace destruction. The septal thickness was seen to be significantly raised in patients with mild interstitial lung disease.

*Xenon transfer contrast* (*XTC*) is an alternative approach to fight the relatively weak hp ^129^Xe signal originating from the dissolved phase through the usage of indirect detection of the dissolved phase in the gas phase [76]. The underlying principle is that hp ^129^Xe exchanges not only from the gas phase to the dissolved phase but also vice versa from the tissue into the alveolar space. Therefore, chemical shift selective destruction of the hp ^129^Xe magnetization (i.e. saturation) in the dissolved phase by 90° pulses can be observed indirectly through a reduction of alveolar hp ^129^Xe gas phase signal. The advantage is that the alveolar signal is much stronger and hence easier to detect. The reduction of the signal is measured in comparison with experiments without chemical shift selective saturation. Since the concept is based on gas exchange, it allows for regional measurement of gas diffusion into the parenchyma.

To obtain spatial information the XTC preparatory sequences are usually combined with FLASH imaging protocol. To further maximize the image contrast the signal associated with the dissolved phase can be inverted rather than suppressed [77,78]. Information is obtained from the decrease of the gas phase signal after multiple exchange times during the XTC sequence as it is proportional to the surface to volume ratio between the lung parenchyma and airspaces. Consequently, the increase of the gas phase signal is indicative of alveolar membrane thickening. With this in mind regional gas exchange has been probed in healthy humans and subjects with COPD [78]. Reduced surface area that corresponded to destruction of the airspaces and septal wall thickening resulted in distinctive contrast in XTC images.

### 6.1 *In vivo* delivery of dissolved ^129^Xe as a contrast agent

**Fig. 8:**
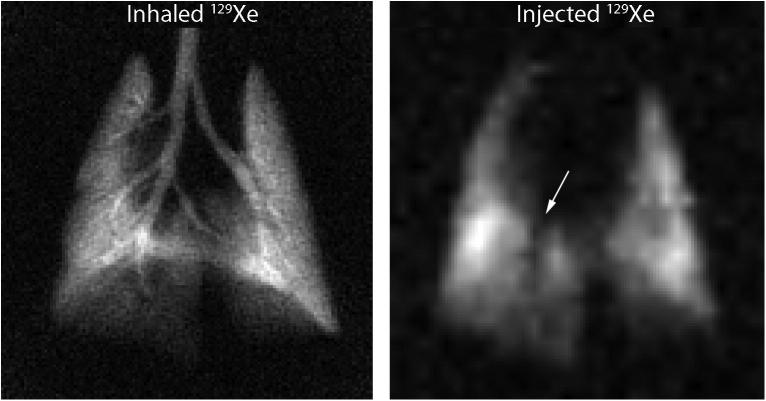
Gas phase image from rat lung with directly inhaled hp ^129^Xe and delivered by injection of hp ^129^Xe in saline solution. The injected image shows a signal void corresponding to the right main stem bronchus (arrow). Reprinted with permission from Driehuys et al. Radiology, 2009; 252:390. © 2009 Radiological Society of North America.

As ^129^Xe is reasonably soluble in saline solution, it can also be added to physiological solutions and then injected into the blood stream [79]. The *T*_1_ relaxation time of hp ^129^Xe is in excess of 60 s in saline solution, reduces to 13 s in oxygenated blood, and is further shortened in deoxygenated blood [80,81]. After intravenous injections, the hp ^129^Xe is delivered through the blood stream (i.e. via perfusion) and subsequent diffusion through the lung parenchyma into the alveolar gas phase. A lack of alveolar hp ^129^Xe indicates either inhibited perfusion or thickening of the lung parenchyma. The MR contrast in these images is thus indicative to vital lung function such as perfusion and blood–gas exchange. It is instructive to compare these images with ventilation sensitive MRI where hp ^129^Xe is delivered through direct inhalation (see Fig. 8). The intravenous delivery method suffers however from low xenon signal intensity and is limited by the volume of saline that can safely be infused *in vivo*. The use of hollow-fiber membranes has however allowed continuous delivery of xenon [82] and thus has resulted in improved detection of the hp ^129^Xe dissolved phase in the lungs [83].

Dissolved phase hp ^129^Xe imaging can also be applied *in vivo* to non-respiratory body systems and adds a novel complementary investigative tool for neuroimaging. The first spectra and chemical shift images using inhaled hp ^129^Xe delivered to the brain through the bloodstream were acquired by Swanson et al. [84]. Intra-arterial deliveries of hp ^129^Xe dissolved in lipid emulsions and gas micro-bubbles were utilized to improve transport to the cerebral circulation but image quality was again limited by the quantities and the time-frame for hp ^129^Xe delivery [85,86], particularly as the longitudinal relaxation time of ^129^Xe dissolved in the rat brain *in vivo* was thought to be of a similar order to that required for uptake by cerebral tissues [87]. After correction for *in vivo* SNR levels, rat brain *T*_1_ times were found to be 15.3 ± 1.2 s and 16.2 ± 0.9 s using two separate protocols [88]. Meanwhile Kershaw, Nakamura and coworkers independently helped to unravel the complex dissolved phase spectra from the rat brain [89,90]. The group found that a complex system of five peaks was reliably resolvable after meticulous shimming. The group demonstrated that the dominant peak arises from brain tissue, presumably from the grey matter (cortex), whilst another lesser peak is likely attributable to the white matter.

**Fig. 9:**
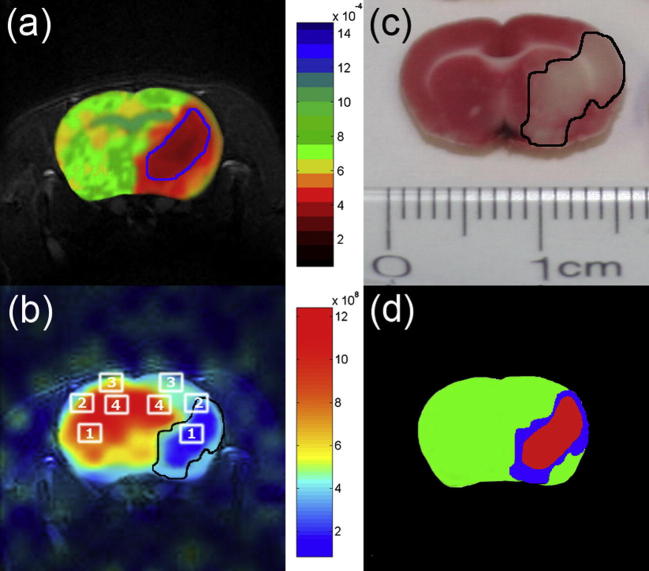
(a) Proton apparent diffusion coefficient (ADC) map image post right middle cerebral artery occlusion. Ischemic core (absent perfusion) is indicated by ADC values <5.30 × 010^−4^ mm^2^/s (circled by blue line). (b) Corresponding hp ^129^Xe chemical shift image displayed in arbitrary units with the greatest signal originating from healthy brain tissue and an obvious signal void corresponding to the ischemic core. (c) 2,3,5-triphenyltetrazolium chloride (TTC)-stained brain section of the same animal. (d) Tricolor map based on the ADC and TTC images shown in (a) and (c) with healthy brain tissue (green), ischemic core (red) and penumbra (blue). Reprinted with permission from Zhou et al. Nmr Biomed. 2011; 24:173. © 2011 John Wiley and Sons, Inc. (For interpretation of the references to color in this figure legend, the reader is referred to the web version of this article.)

Images of middle cerebral artery occlusions in rats have since been acquired that demonstrate the absence of the dissolved hp ^129^Xe signal in regions with acute ischemia and the poorly perfused surrounding penumbra (Fig. 9) [91]. Moreover, functional brain images produced during painful stimuli in rats displayed enhanced cerebral hp ^129^Xe uptake in areas of the brain that largely corresponded to sensory regions previously identified by proton functional MRI methods [92]. Though ^129^Xe images are of lower spatial and temporal resolution than ^1^H arterial spin labeled (ASL) images, a great correlation between the two techniques adds another delightful perspective for the possible use of hp ^129^Xe in functional brain imaging and diagnosis.

## 7 Towards molecular imaging using functionalized ^129^Xe biosensors and HYPER-CEST

**Fig. 10:**
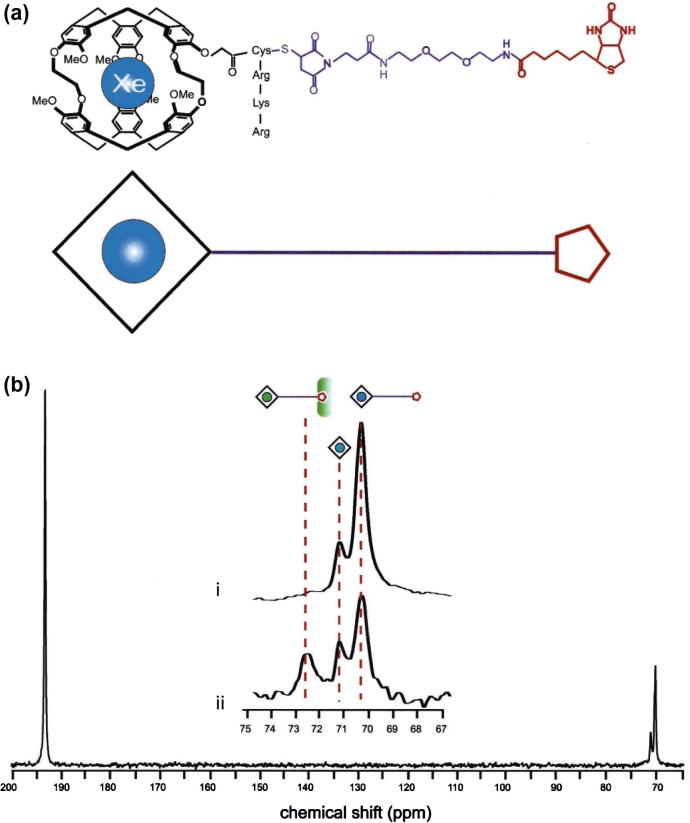
Hp ^129^Xe biosensor approach. (a) Cryptophane-A cage, acting as a xenon encapsulating agent, tethered to a bioactive ligand (red) with a specific binding affinity for avidin. (b) Full spectrum (bottom): actual hp ^129^Xe spectrum of the biosensor in the absence of avidin. The signal at 193 ppm is from xenon in solution (i.e. water) while the signal at 70 ppm corresponds to xenon in the cryptophane-A cage of the biosensor molecule shown in (see inset i). The signal at 71 ppm originates from xenon in cryptophane-A cages without tether. When avidin is added, another signal appears (ii) just below 73 ppm. Adapted figure, printed with permission from Spence et al. P. Ntl. Acad. Sci. USA, 2001; 98:10655. © 2001 National Academy of Sciences, USA. (For interpretation of the references to color in this figure legend, the reader is referred to the web version of this article.)

Molecular imaging, i.e. the detection of the spatial distribution of specific target molecules in an organism provides tremendous opportunities for biomolecular research. The challenge for molecular MRI lies in the inherent complexity of NMR spectra and the low signal intensity typically associated with dilute concentrations of the target molecules. One pathway, that has attracted a great deal of attention, is dynamic nuclear polarization (DNP) of molecules that are isotopically labeled at specific sites, resulting in NMR spectra with high signal intensity and manageable complexity [93]. However, the large chemical shift range of ^129^Xe and the simplicity of typical ^129^Xe NMR spectra opens up an alternative approach to molecular imaging. In 2001, Pines, Wemmer, and co-workers undertook the first step into molecular MRI using hp ^129^Xe [94] and the underlying concept, developed by this group, bears significant potential for future biomedical applications [95,96]. The fundamental idea is, reminiscent of fluorescence labeling, to use bio-sensor molecules that contain bioactive ligands with a specific binding affinity for particular analytes (Fig. 10). In the original work, biotin as a ligand for the protein avidin was used but the concept can be extended from peptide–antigen recognition as shown by Schlund et al. [97], to specific binding to nucleotide targets as demonstrated through *in vitro* recognition of a DNA strand by Berthault and co-workers [98], and to cancer biomarkers as reported by Dmochowski and co-workers [99,100]. Linked via a molecular tether to the specific ligand is an encapsulating agent, such as a cryptophane cage, that can bind a single xenon atom. ^129^Xe bound to the cages will resonate at a chemical shift that is distinct from the resonance of the xenon dissolved in the solvent and that is specific for the type of encapsulating cage used. Further, the ^129^Xe chemical shift observed in the cage changes slightly between protein-bound and unbound biosensors, presumably because of distortions in the cage structure. The cages are required to have a high binding affinity for xenon but also need to allow for fast exchange with the hyperpolarized xenon atoms in solution, yet slow enough to prevent coalescence of the chemical shift differences. Useful exchange rates should therefore be somewhere in the 10–100 Hz regime. Cryptophanes [101,102] are the most widely studied xenon encapsulating molecules as they have a high binding affinity, allow for sufficient exchange, and provide a large (chemical shift) shielding for the encapsulated xenon atoms due to the presence of aromatic rings. Particularly useful properties for biomedical applications are that the cages can be chemically modified and that several water-soluble cryptophanes with large xenon binding affinity have been synthesized [103–105].

For hyperpolarized ^129^Xe MR bio-detection, the biosensor molecule is administered long before the hp ^129^Xe is transferred to the organism. Hp ^129^Xe can be delivered into blood stream via injection [106] or simply through inhalation. Relaxation within the cages is on the order of many tens of seconds and thus will interfere little with the detection scheme. Since different cages with distinct ^129^Xe chemical shifts and different binding moieties can be used concurrently, the simultaneous recognition of different target molecules, i.e. multiplexing, is possible [94,107].

### 7.1 HYPER-CEST

The scheme described above allows for MRI detection of (multiple) immobilized biosensors bound to targets present in a stationary matrix. Since the hp ^129^Xe can be delivered in excess, biosensor detection in the micro-molar regime is possible. The sensitivity can be significantly increased further through an indirect detection method developed by Pines and co-workers [108]. HYPER-CEST is a combination of CEST (chemical exchange saturation transfer) with hp ^129^Xe and is reminiscent of the concept described for XTC above. Chemical shift selective irradiation at the ^129^Xe frequency of the bound xenon is applied to destroy the hyperpolarized state. Chemical exchange between bound xenon and xenon in the bulk solution (for instance blood) then leads to a depletion of the bulk solution hp ^129^Xe signal as long as the irradiation is applied. The signal reduction is indicative of the biosensor presence and therefore of the target molecule. Because the ^129^Xe signal arising from the bulk solution is much stronger than that from the bound xenon, and because the depletion can be ‘accumulated’ over time, HYPER-CEST allows for nano-molar sensitivity. The technique requires however, that the hp ^129^Xe polarization level in the solution does not significantly fluctuate due to other causes.

Additional ways to boost sensitivity for xenon-biosensors are in the usage of dendrimer–cryptophane supramolecular constructs [109] and viral capsid scaffolds [110] that both increase the number of cages per target molecule. Further, functionalized zeolite nano-particles have also been explored as potential biosensors [111]. The advantage of these particles is that they may accommodate a copious amount of xenon atoms leading to a stronger directly detected signal.

## 8 Remote detection

The concept of gas MRI can be extended by a remote detection scheme developed by Pines and co-workers [112] where the excitation coil and pulsed magnetic field gradient coils are completely separated in space from the detection coil. In this scheme, hp ^129^Xe is delivered to the sample region where the excitation and encoding take place. The hp ^129^Xe is then transferred to a distant detection region where the encoded information is read out with a higher sensitivity than what would be possible in the sample region. In its most basic form, this scheme does not have a direct dimension (such as frequency encoding) and requires point-by-point measurement of the encoded phase for all dimensions. The long hp ^129^Xe *T*_1_ relaxation facilitates the experiments as the encoded information is stored as “magnetization”, despite the 50% signal loss associated with the use of a storage pulse analogue to that in stimulated echo sequences. After storage, the information transport can take place via gas flow to a distant detection cell where the encoded information is read after another excitation pulse. Gas mixing during transport typically does not cause problems for the read out of the stored spatial information as long as there is no mixing between the individual point-by-point experiments. The main advantage of this technique is that the encoding and detection regions can be independently optimized, the former for versatility of the encoding and the latter can be optimized for sensitivity. An increased sensitivity could be achieved using a coil that may be smaller than the actual sample leading to an improved filling factor and the detection field strength may be higher than in the sample region. This scheme allows for samples to be used that could not be measured sensitively in an NMR experiment, such as a magnetic porous material. The mobile phase can be encoded within this porous substance while the detection will be spatially removed from the material. Alternatively, detection methods that are not based on Faraday inductive detection may be employed to provide higher sensitivity [113,114].

**Fig. 11:**
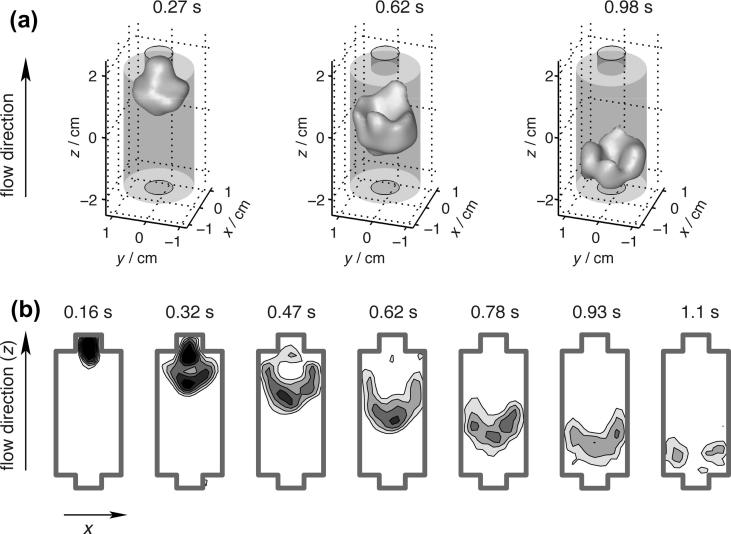
Remotely detected images of gas flow through the rock sample for various arrival times (i.e. time between encoding and detection) as indicated above each image. As the fluid flows through a sample, the nuclear spin magnetization is modulated by RF pulses and magnetic field gradients to encode its spatial coordinates. After leaving the sample, the ^129^Xe magnetization is recorded when it arrives at the detection coil. (a) 3D representation, (b) time of flight x–z images with varying detection times after encoding. Reprinted figure with permission from Granwehr et al. Phys. Rev. Lett., 2005; 95:075503-3. © 2005 American Physical Society.

While remote detection does not contain a direct spectral or imaging dimension, the arrival of the encoded gas can be monitored transiently, thereby retrieving time-of-flight information in a direct dimension. This enables visualization of flow and diffusion through, for example, a porous rock sample [115] or through microfluidic devices [116,117]. The gas from various regions (and therefore the encoded information) may arrive at different times (of flight) as shown in Fig. 11. The ^129^Xe chemical shift can also be utilized in remotely detected MRI to separate between different environments of the gas flowing through porous systems [118]. Perhaps most interesting for biomedical applications, is that the remote detection concept can be extended to MRI of dissolved xenon with detection after extraction from the liquid to the gas phase via membranes [119]. Remote detection of hp gases can also be utilized for relaxation measurements and may be particularly useful for field dependent relaxometry studies [120].

As a note of caution, remote detection suffers from the absence of a direct dimension (i.e. there is no frequency encoding) because the information has to be collected point by point. For instance, a 64 × 64 two-dimensional MR image requires a minimum of 4096 scans as opposed to 64 scans for directly detected MRI. On the other hand, time-of-flight information can be recorded transiently, which facilitates a different type of direct dimension than in conventional Fourier imaging techniques. Therefore continuous flow type of experiments are probably most practical for remote detection. Further, remote detection also requires that fluctuations in the gas delivery and spin polarization are kept at a minimum although calibration experiments can sometimes correct for such fluctuations [120].

## 9 Hp ^83^Kr MRI

One of the advantages of hp ^129^Xe MRI is the associated large chemical shift that is indicative of small distortions in xenon electron cloud and is therefore a valuable probe of the atomic and molecular surrounding. The ^129^Xe chemical shift is unsurpassed by any other stable noble gas isotope. However, ^131^Xe, another NMR active and stable xenon isotope, has a nuclear spin I = 3/2 and therefore possesses a nuclear electric quadrupole moment that can also serve as a fairly sensitive detector of atomic electron cloud distortions. It is therefore a much more sensitive probe for noble gas–surface interactions than the ^129^Xe chemical shift and the isotope can provide surface sensitive MRI contrast [121]. Unfortunately, even gas phase collisions cause rapid quadrupolar driven relaxation that leads to short ^131^Xe *T*_1_ times and therefore rapid decay of the hyperpolarized state [29]. However, another noble gas isotope with a nuclear electric quadrupole moment, namely ^83^Kr, typically displays a slower quadrupolar relaxation compared to ^131^Xe because of krypton’s smaller electron cloud and because of its larger nuclear spin *I* = 9/2. The remarkably long ^83^Kr gas-phase *T*_1_ times of up to several hundred seconds at ambient pressure allow for hyperpolarization up to *P* = 26%. Because of dilution with other gases, the best currently available apparent (i.e. effective) polarization is 3% [31].

**Fig. 12:**
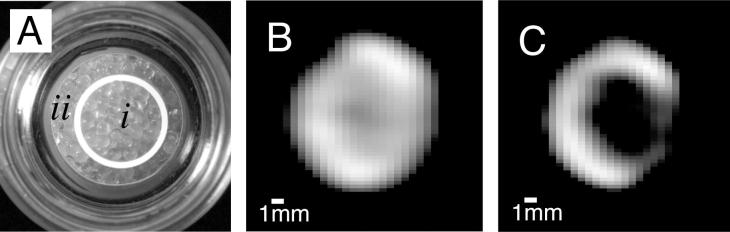
(A) Photograph of a sample containing 1.0 mm glass beads with a siliconized, hydrophobic surface in compartment (*i*) and an untreated, hydrophilic surface in compartment (ii). (B) MRI of gas phase hp ^83^Kr shortly after transfer into the sample. (C) Hp ^83^Kr MRI as in (B) but after additional 6 s delay time, showing a surface sensitive MRI contrast. The ^83^Kr quadrupolar relaxation caused by surface interactions leads to *T*_1_ = 9 s in the hydrophobic region (i) and to *T*_1_ = 35 s in the hydrophilic region (ii). Adapted figure, printed with permission from Pavlovskaya et al. P. Ntl. Acad. Sci. USA 2005; 102:18278 © 2005 National Academy of Sciences, USA.

The quadrupolar longitudinal ^83^Kr relaxation can be utilized for MR studies of surrounding surfaces since it is susceptible to the surface-to-volume ratio, surface hydration, and surface temperature [28]. Hyperpolarized (hp) ^83^Kr has been shown to provide *T*_1_ relaxation weighted MRI contrast that is highly sensitive to the surface chemistry in low surface-to-volume model systems. Fig. 12 provides an example of surface sensitive contrast in hp ^83^Kr gas phase MRI. Hp ^83^Kr NMR relaxation measurements of excised but actively ventilating rat lungs have been used recently to study *T*_1_ relaxation as a function of lung inflation [122]. The longitudinal ^83^Kr relaxation in the distal airways and the respiratory zones was found to be independent of the lung inhalation volume and highly reproducible between different specimens. The *T*_1_ relaxation times ranged between 1.0 and 1.3 s and should be long enough for *in vivo* usage of hp ^83^Kr MRI with rats that typically breathe at a rate of around 1 Hz while anesthetized. Further, the relaxation should be slower in larger animals if surface to volume ratio decrease with larger alveoli diameters. A spatially resolved relaxation study may provide insights into alveolar recruitment and may also be indicative of diseases that affect lung surface to volume ratios or the chemical composition of the lung surface, for instance through alterations of the surfactant concentration. Recent improvements in SEOP have increased the hp ^83^Kr signal intensity significantly [31] and enabled coronal lung FLASH MRI of excised rat lungs in unpublished, preliminary studies.

As some final notes, krypton is obtained from air liquefaction at approximately tenfold lower cost than xenon – i.e. on the order of US$ 0.80–1.00 per liter gas. Unfortunately, due to rare demand the costs for isotopically enriched ^83^Kr is currently about US$ 5000 per liter. At ambient pressure, krypton has no anesthetic properties [45]. The isotope ^83^Kr has a natural abundance of 11.5% and its NMR resonance frequency in the gas phase at ambient pressure and temperature is 3.85 MHz at 2.35 T magnetic field strength. As a consequence of its extremely low gyromagnetic ratio, the ^83^Kr T_2_ relaxation times are typically much longer than that of ^129^Xe. Furthermore, due to its low *γ*, the ^83^Kr *T*_1_ relaxation in rat lungs is not affected by the presence of up to 40% paramagnetic oxygen [122]. Note that although hp ^83^Kr may dissolve in many tissues, the useful signal associated with its dissolved phase is lost owing to fast quadrupolar relaxation.

## 10 Conclusions

Recent and currently ongoing advances in the hp ^129^Xe production, in optimization of MRI methods, and in regulatory compliance associated with clinical hp ^129^Xe usage may allow for hp ^129^Xe MRI to substitute some of the ^3^He MRI applications in the intermediate future. However, hp ^129^Xe MRI also provides complementary information to existing ^3^He techniques because xenon tissue solubility and the ^129^Xe chemical shift allow diagnostic studies of lungs in health and disease as shown in elegant experiments by a number of groups. The advent of biosensors promises to extend the scope of hp ^129^Xe MRI towards molecular imaging.

Although the materials science and engineering applications for hp ^129^Xe have predominately been in NMR spectroscopy, hp ^129^Xe MRI should also be attractive for the respective communities. The potential significance of hp ^129^Xe applications within non-biomedical research fields is for non-invasive spatially resolved transport measurements. These applications may involve remote detection schemes that allow for measurements in materials environments that usually do not allow for straightforward NMR detection.

Of the quadrupolar noble gas isotopes, ^83^Kr is most likely find usage as a surface sensitive MRI contrast agent. The currently available polarization levels allow for proof of principle studies and first applications in pulmonary imaging. The highest benefit of hp ^83^Kr MRI contrast will likely be obtained in conjunction with the higher resolution of hp ^129^Xe MRI measurements.
